# Progressive fibrosing interstitial lung disease: prevalence and clinical outcome

**DOI:** 10.1186/s12931-021-01879-6

**Published:** 2021-10-31

**Authors:** Byoung Soo Kwon, Jooae Choe, Eun Jin Chae, Hee Sang Hwang, Yong-Gil Kim, Jin Woo Song

**Affiliations:** 1 Division of Pulmonary and Critical Care Medicine, Department of Internal Medicine, Seoul National University Bundang Hospital, Seongnam-S, Gyeonggi-Do Republic of Korea; 2 Department of Radiology, University of Ulsan College of Medicine, Asan Medical Centre, Seoul, Republic of Korea; 3 Department of Pathology, University of Ulsan College of Medicine, Asan Medical Centre, Seoul, Republic of Korea; 4 Department of Rheumatology, University of Ulsan College of Medicine, Asan Medical Centre, Seoul, Republic of Korea; 5 Department of Pulmonology and Critical Care Medicine, University of Ulsan College of Medicine, Asan Medical Centre, 88 Olympic-ro 43-gil, Songpa-gu, Seoul, Republic of Korea

**Keywords:** Phenotype, Interstitial lung disease, Outcome, Prevalence, Progressive

## Abstract

**Background:**

The progressive fibrosing (PF) phenotype of interstitial lung disease (ILD) is characterised by worsening respiratory symptoms, lung function, and extent of fibrosis on high-resolution computed tomography. We aimed to investigate the prevalence and clinical outcomes of PF-ILD in a real-world cohort and assess the prognostic significance of the PF-ILD diagnostic criteria.

**Methods:**

Clinical data of patients with fibrosing ILD other than idiopathic pulmonary fibrosis (IPF) consecutively diagnosed at a single centre were retrospectively reviewed. A PF phenotype was defined based on the criteria used in the INBUILD trial.

**Results:**

The median follow-up duration was 62.7 months. Of the total of 396 patients, the mean age was 58.1 years, 39.9% were men, and rheumatoid arthritis-ILD was the most common (42.4%). A PF phenotype was identified in 135 patients (34.1%). The PF-ILD group showed lower forced vital capacity and total lung capacity (TLC) than the non-PF-ILD group. The PF-ILD group also showed poorer survival (median survival, 91.2 months vs. not reached; *P* < 0.001) than the non-PF-ILD group. In multivariable Cox analysis adjusted for age, DL_CO_, HRCT pattern, and specific diagnosis, PF phenotype was independent prognostic factor (hazard ratio, 3.053; *P* < 0.001) in patients with fibrosing ILD. Each criterion of PF-ILD showed similar survival outcomes.

**Conclusions:**

Our results showed that approximately 34% of patients with non-IPF fibrosing ILD showed a progressive phenotype and a poor outcome similar to that of IPF, regardless of the diagnostic criteria used.

**Supplementary Information:**

The online version contains supplementary material available at 10.1186/s12931-021-01879-6.

## Background

Interstitial lung disease (ILD) encompasses heterogeneous disorders with various clinical courses [1]. Idiopathic pulmonary fibrosis (IPF) is a prototype of progressive fibrosing ILD with a poor prognosis. However, fibrosing ILDs other than IPF, such as idiopathic nonspecific interstitial pneumonia (iNSIP), fibrotic hypersensitivity pneumonitis (HP), and autoimmune ILD, also have a progressive phenotype, manifesting as worsening of dyspnoea, decline in lung function, and increased extent of fibrosis on high-resolution computed tomography (HRCT), with high morbidity and mortality [2]. Recently, a subset of non-IPF fibrosing ILDs with a progressive course despite conventional treatment has been referred to as progressive fibrosing ILD (PF-ILD) [3]. From a pathobiological perspective, it was reported that the mechanisms of fibrosis in non-IPF ILDs, including rheumatoid arthritis (RA)-ILD and HP, have commonalities with IPF such as *MUC5B* single nucleotide polymorphism and short telomere length [3–6]. Moreover, the prognosis of PF-ILD was reported as comparable to that of IPF [7]. Brown et al., in the analysis of two randomised controlled trials involving IPF and PF-ILD (INPULSIS [8] and INBUILD [9]), reported that lung function decline and mortality rates were similar in both placebo groups of the IPF and PF-ILD cohorts [7].

The proportion of a progressive phenotype in non-IPF ILDs was reported to be approximately 30% in a real-world [2, 10]. According to a survey of ILD experts and a US insurance claim database, 18%–32% of patients with non-IPF ILDs were estimated to develop a progressive phenotype [2]. Similarly, Nasser et al. [10], in an analysis of 617 patients with ILD other than IPF, reported that 27.2% of patients met the criteria for disease progression defined in the INBUILD trial [9]. However, although interest in PF-ILD is increasing, there has been no consensus definition of PF-ILD yet. In addition, data on PF-ILD are still limited, such as its proportion among non-IPF fibrosing ILD, risk factors, and clinical outcomes in a real-world cohort. Therefore, the aim of this study was to evaluate the prevalence, risk factors, and survival of PF-ILD, and to assess the prognostic value of various PF-ILD criteria in patients with non-IPF fibrosing ILD.

## Materials and methods

### Study population

**Fig. 1:**
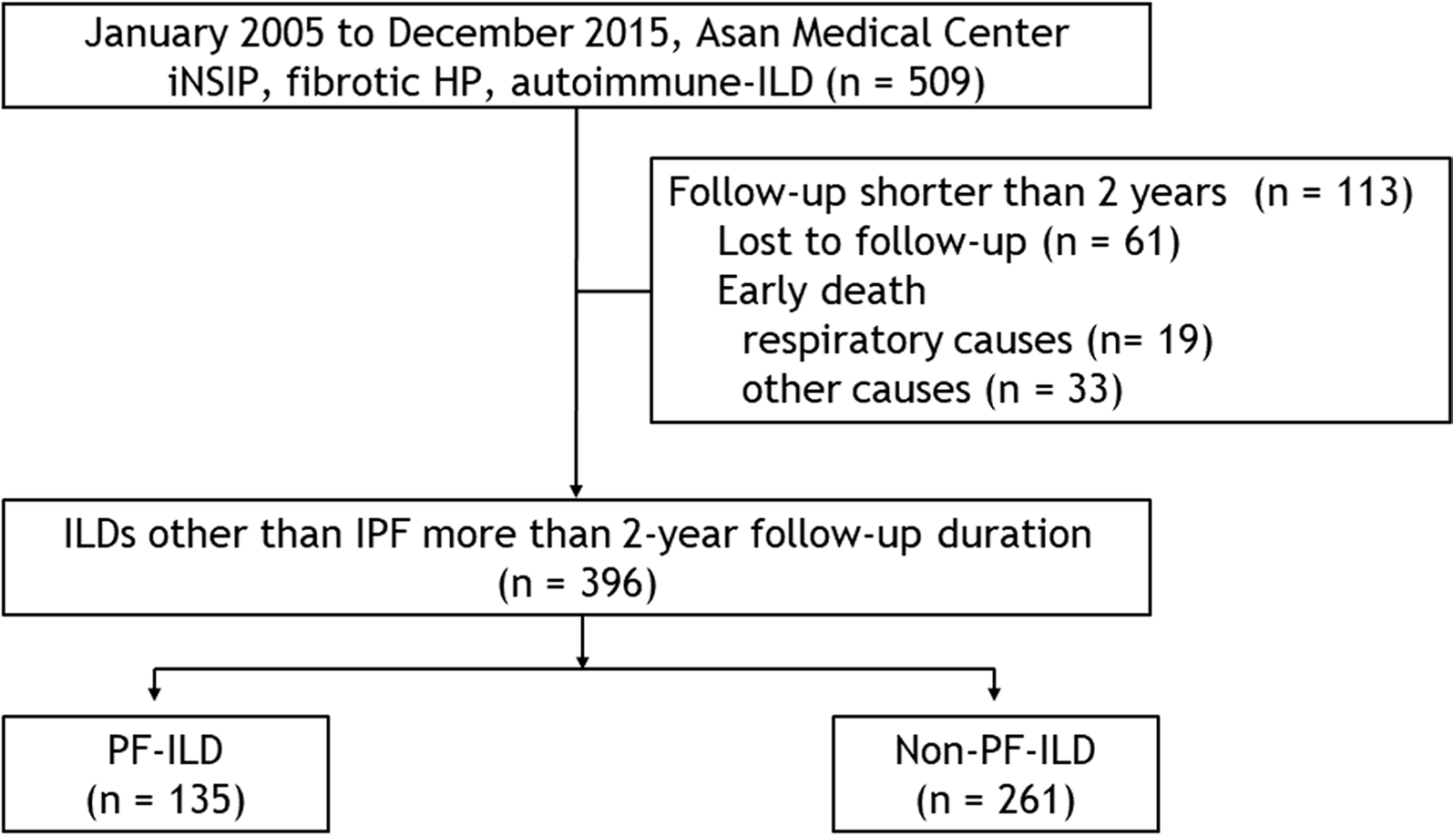
Study flow chart. *ILD* interstitial lung disease, *IPF* idiopathic pulmonary fibrosis, *PF* progressive fibrosing, *iNSIP* idiopathic nonspecific interstitial pneumonia, *HP* hypersensitivity pneumonitis

Between January 2005 and December 2015, patients with iNSIP (n = 98; all biopsy confirmed), fibrotic HP (n = 76; all biopsy confirmed), and autoimmune ILDs (n = 335; biopsy confirmed cases = 85), who were consecutively diagnosed at Asan Medical Centre, Seoul, Republic of Korea, were identified and constituted the non-IPF fibrosing ILD cohort (n = 509). Autoimmune ILDs involved RA (n = 214), systemic sclerosis (SSc, n = 67), and Sjögren syndrome (SJS, n = 54). Within the cohort, 113 patients who were followed up for < 2 years were excluded. In all, 396 patients were included in the analysis (Fig. 1).

All patients with iNSIP and fibrotic HP met the diagnostic criteria of the American Thoracic Society (ATS) project [11] and the ATS/Japanese Respiratory Society/Latin American Thoracic Association clinical practice guidelines [12], respectively. All patients with autoimmune ILDs met the American College of Rheumatology classification criteria for RA, SSc, and SJS [13–15]. All diagnoses were confirmed by multidisciplinary discussion. This study was approved by the Institutional Review Board of Asan Medical Centre (IRB 2020-0705), and informed consent was waived due to the retrospective design.

### Data collection

Clinical and survival data for all patients were retrospectively obtained from medical records, telephone interviews, and/or records of the National Health Insurance of Korea. Pulmonary function tests (PFTs), including forced vital capacity (FVC), diffusing capacity of the lung for carbon monoxide (DL_CO_), and total lung capacity (TLC), were measured according to ATS/European Respiratory Society (ERS) recommendations [16–18]. PFT results are presented as percentages of normal predicted values.

Records of follow-up visits (usually every 3–6 months) and hospitalisations were reviewed to identify the development of complications such as pneumonia, acute exacerbation (AE), pneumothorax, pulmonary embolism, and pulmonary hypertension (PH). Acute exacerbation was defined according to the criteria suggested by Collard et al. [19]. PH (intermediate to high probability) was defined as a maximal tricuspid regurgitation velocity of ≥ 2.9 m/s according to the 2015 European Society of Cardiology/ERS guidelines [20].

### Definition of PF-ILD

The definition of PF-ILD, based on the INBUILD criteria [9], is fibrosis extent more than 10% of the total lung on HRCT, along with one of the following criteria within the 24 months after diagnosis despite standard of care: (i) a relative decline in FVC ≥ 10%; (ii) a relative decline in FVC of 5%–10% and worsening of respiratory symptoms or increased extent of fibrosis on HRCT; or (iii) worsening of respiratory symptoms and increased extent of fibrosis on HRCT. Worsening of respiratory symptoms was considered as an increase of one or more points in the modified Medical Research Council dyspnoea scale.

The prognostic value of three reported sets of PF-ILD diagnostic criteria were also evaluated [21–23]. Cottin et al. defined PF-ILD as developing any of the following within 2 years of diagnosis: a relative decline in FVC ≥ 10%; a relative decline in DL_CO_ ≥ 15%; worsening of symptoms or radiological appearance and relative decline in FVC from 5% up to < 10% [21]. Behr et al. defined progression as an absolute annual decline in FVC ≥ 5% of predicted value within 6–24 months of diagnosis [22]. George et al. defined PF-ILD as developing any of the following within 2 years of diagnosis despite treatment: a relative decline in FVC ≥ 10%; a relative decline in FVC ≥ 5% with decline in DL_CO_ ≥ 15%; a relative decline in FVC ≥ 5% with worsening of symptoms or radiological appearance; worsening of symptoms and increased extent of fibrosis [23].

### HRCT evaluation

HRCT scans were obtained in accordance with standard protocols at full inspiration without contrast enhancement. The HRCT images were independently reviewed by two thoracic radiologists (JC, and EJC) blinded to the clinical and pathologic information. HRCT images acquired at diagnosis and 2 years after diagnosis were reviewed, and increased extent of fibrosis on HRCT was visually assessed with side-by-side comparison. The thoracic radiologists also determined whether the fibrosis involved > 10% of the total lung. HRCT patterns were classified as either usual interstitial pneumonia (UIP)-like or non-UIP-like. A UIP-like pattern was defined as reticular abnormality and traction bronchiectasis with or without honeycombing, a basal and peripheral predominance, and absence of atypical features such as extensive ground-glass opacity, nodules, or consolidation [9]. Disagreement between the readers was resolved via a consensus.

### Statistical analysis

All values are expressed as mean ± standard deviation for continuous variables and as percentages for categorical variables. The Student’s *t*-test was used for continuous data, and Pearson’s chi-square test or Fisher’s exact test was used for categorical data. Risk factors for PF-ILD were evaluated by logistic regression analysis. Survival was examined using the Kaplan–Meier analysis and log-rank test. The survival time was analysed from initial diagnosis to the date of death or censoring (lost to follow-up or 31 December 2018). A Cox proportional hazards model was used to determine variables related to survival. Variables with a *P*-value < 0.1 in the unadjusted analysis were entered into the multivariable model and selected using the backward log-likelihood ratio statistics method. *P*-values < 0.05 were considered statistically significant. Statistical analyses were performed using IBM SPSS version 24 (SPSS, Inc., Chicago, IL, USA) and Prism version 5 (GraphPad, San Diego, CA, USA).

## Results

### Prevalence

The median patient follow-up period was 62.7 months (interquartile range, 42.6–89.4 months). The mean patient age was 58.1 years, and 39.9% were men. Among all patients, autoimmune ILD was the most common diagnosis (67.7%), followed by iNSIP (19.2%) and fibrotic HP (13.1%) (Table 1). Among the autoimmune ILDs, RA-ILD was the most common (62.7%), followed by SSc-ILD (20.1%) and SJS-ILD (17.2%).

**Table 1:** Comparison of baseline characteristics between the PF-ILD and non-PF-ILD groups among patients with fibrosing ILD

	Total	PF-ILD	Non-PF-ILD	*P*-value
Number of patients	396	135	261	
Age, years	58.1 ± 10.6	58.7 ± 10.1	57.9 ± 10.8	0.474
Male	158 (39.9)	58 (43.0)	100 (38.3)	0.388
BMI, kg/m^2^	23.8 ± 3.3	24.3 ± 3.6	23.6 ± 3.1	0.074
Ever-smokers	163 (41.2)	56 (41.5)	107 (41.0)	> 0.999
ILD subtype				0.328
iNSIP	76 (19.2)	20 (14.8)	56 (21.5)	
HP	52 (13.1)	29 (21.5)	23 (8.8)	
RA-ILD	168 (42.4)	58 (43.0)	110 (42.1)	
SSc-ILD	54 (13.6)	18 (13.3)	36 (13.8)	
SJS-ILD	46 (11.6)	10 (7.4)	36 (13.8)	
PFT, % predicted				
FVC	73.3 ± 16.6	70.5 ± 16.5	74.7 ± 16.5	0.016
DL_CO_	62.4 ± 18.0	60.0 ± 16.7	63.6 ± 18.6	0.059
TLC	74.9 ± 14.8	71.7 ± 13.6	76.6 ± 15.1	0.002
HRCT pattern				0.750
UIP-like pattern	181 (45.7)	60 (44.4)	121 (46.4)	
Non-UIP-like pattern	215 (54.3)	75 (55.6)	140 (53.6)	
Treatment				0.078
Steroid ± IM^a^	365 (92.2)	129 (95.6)	236 (90.4)	
No treatment	31 (7.8)	6 (4.4)	25 (9.6)	

Data are expressed as mean ± standard deviation or number (%), unless otherwise indicated. ^a^Mycophenolate mofetil (n = 151), azathioprine (n = 110)

*PF* progressive fibrosing, *ILD* interstitial lung disease, *BMI* body mass index, *iNSIP* idiopathic nonspecific interstitial pneumonia, *HP* hypersensitivity pneumonitis, *PFT* pulmonary function test; *FVC* forced vital capacity, *DL*_*CO*_ diffusing capacity of the lung for carbon monoxide, *TLC* total lung capacity, *HRCT* high-resolution computed tomography, *UIP* usual interstitial pneumonia, *IM* immunosuppressant

**Fig. 2:**
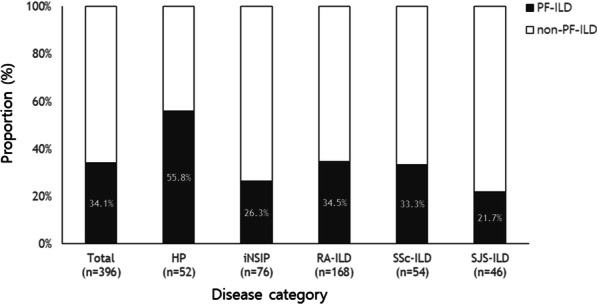
Prevalence of PF-ILD. *PF* progressive fibrosing, *ILD* interstitial lung disease, *HP* hypersensitivity pneumonitis, *iNSIP* idiopathic nonspecific interstitial pneumonia, *RA* rheumatoid arthritis, *SSc* systemic sclerosis, *SJS* SjÖgren syndrome

A progressive phenotype was identified in 135 patients (34.1%). PF-ILD was the most frequently identified in fibrotic HP (55.8%), followed by autoimmune ILD (32.1%) and iNSIP (26.3%) (Fig. 2). Among the autoimmune ILDs, the proportions of PF-ILD in patients with RA, SSc, and SJS were 34.5%, 33.3%, and 21.7%, respectively. There was no difference in prevalence according to the HRCT pattern (UIP-like: 33.1% vs. non-UIP-like: 34.9%, *P* = 0.717). The numbers of patients who met each PF-ILD criterion were 86 (63.7%, relative decline in FVC ≥ 10% [criterion i]), 21 (15.6%, relative decline in FVC of 5%–10% and worsening of respiratory symptoms or increased extent of fibrosis [criterion ii]), and 28 (20.7%, worsening of respiratory symptoms and increased extent of fibrosis [criterion iii]).

### Risk factors

The PF-ILD group had lower lung function parameters (FVC and TLC) than the non-PF-ILD group (Table 1).

In the unadjusted logistic analysis, fibrotic HP and lower lung function parameters (FVC and TLC) were significant factors predictive of PF-ILD. In the multivariable analysis, fibrotic HP (odds ratio [OR], 3.763; 95% confidence interval [CI], 1.740–8.138; *P* = 0.001) and lower FVC (OR, 0.982; 95% CI, 0.969–0.996; *P* = 0.011) remained as independent predictive factors for PF-ILD (Table 2). In the subgroup analysis according to ILD subtypes, lower lung function parameters (FVC, TLC, or DLco) were also the risk factors for PF-ILD in patients with HP and autoimmune ILD (Additional file 1: Table S1).

**Table 2:** Risk factors for PF-ILD in patients with fibrosing ILD assessed using logistic regression analysis

	Unadjusted		Multivariable	
	Odds Ratio (95% CI)	*P*-value	Odds Ratio (95% CI)	*P*-value
Age	1.007 (0.988–1.027)	0.473		
Male	1.213 (0.795–1.850)	0.317		
BMI	1.059 (0.994–1.128)	0.076	–	–
Ever-smokers	1.020 (0.669–1.556)	0.926		
ILD subtype		0.004		0.003
iNSIP	1		1	
HP	3.530 (1.670–7.462)	0.001	3.763 (1.740–8.138)	0.001
RA-ILD	1.476 (0.809–2.694)	0.204	1.870 (0.999–3.502)	0.050
SSc-ILD	1.400 (0.653–3.000)	0.387	1.407 (0.643–3.083)	0.393
SJS-ILD	0.778 (0.327–1.851)	0.570	0.778 (0.314–1.925)	0.587
FVC	0.985 (0.972–0.997)	0.017	0.982 (0.969–0.996)	0.011
DL_CO_	0.989 (0.977–1.000)	0.060	–	–
TLC^a^	0.977 (0.963–0.992)	0.003		
UIP-like pattern	0.926 (0.610–1.405)	0.717		
Steroid ± IM	2.278 (0.911–5.695)	0.078	–	–

^a^TLC was excluded in the multivariable analysis due to close correlation with FVC (r = 0.898, *P* < 0.001)

*CI* Confidence interval, *PF* progressive fibrosing, *ILD* interstitial lung disease, *BMI* body mass index, *iNSIP* idiopathic nonspecific interstitial pneumonia, *HP* hypersensitivity pneumonitis, *RA* rheumatoid arthritis, *SSc* systemic sclerosis, *SJS* SjÖgren syndrome, *FVC* forced vital capacity, *DL*_*CO*_ diffusing capacity of the lung for carbon monoxide, *TLC* total lung capacity, *UIP* usual interstitial pneumonia, *IM* immunosuppressant

### Survival

**Fig. 3:**
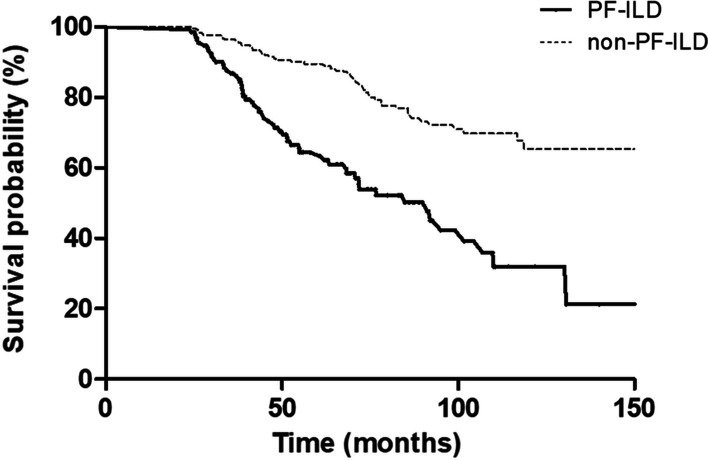
Comparison of survival curves between the PF-ILD and non-PF-ILD groups among patients with fibrosing ILD. *PF* progressive fibrosing, *ILD* interstitial lung disease

During follow-up, 110 patients (27.8%) died. The PF-ILD group showed poorer survival (median survival, 91.2 months vs. not reached; *P* < 0.001) than the non-PF-ILD group (Fig. 3). The 5-year survival rates of the PF-ILD and non-PF-ILD groups were 64.5% and 89.5%. This survival difference was also consistent across each ILD subtype (iNSIP, not reached in the both groups, *P* = 0.080; HP, median survival, 84.9 months vs. not reached; *P* = 0.048; autoimmune ILDs, median survival 91.2 months vs. not reached, *P* < 0.001, Additional file 1: Figs. S1). In the unadjusted Cox analysis, PF-ILD was a significant prognostic factor for mortality in patients with fibrosing ILD, along with older age, lower DL_CO,_ and a UIP-like pattern on HRCT (Table 3). In the multivariable analysis, PF-ILD was an independent prognostic factor (hazard ratio [HR], 3.053; 95% CI, 2.066–4.512; *P* < 0.001) in patients with fibrosing ILD, along with older age, and lower DL_CO_ (Table 3). In the subgroup analysis, PF-ILD was also a prognostic factor for mortality in autoimmune ILD (Additional file 1: Table S2). Among the patients with PF-ILD, older age (HR, 1.072; 95% CI, 1.037–1.109; *P* < 0.001) and lower DL_CO_ (HR, 0.984, 95% CI, 0.967–1.000; *P* = 0.049) were identified as statistically significant prognostic factors for mortality in the multivariable Cox analysis (Additional file 1: Table S3).

**Table 3:** Prognostic factors for mortality in patients with fibrosing ILD assessed using a Cox proportional hazards model

	Unadjusted		Multivariable	
	Hazard Ratio (95% CI)	*P*-value	Hazard Ratio (95% CI)	*P*-value
Age	1.069 (1.047–1.091)	< 0.001	1.075 (1.050–1.100)	< 0.001
Male	1.312 (0.901–1.909)	0.157		
BMI	1.025 (0.968–1.086)	0.401		
Ever-smokers	1.137 (0.780–1.657)	0.506		
ILD subtype		0.078	–	–
iNSIP	1			
HP	1.874 (0.955–3.678)	0.068		
RA-ILD	1.325 (0.769–2.281)	0.311		
SSc-ILD	0.603 (0.250–1.455)	0.260		
SJS-ILD	1.087 (0.528–2.241)	0.820		
FVC	0.995 (0.984–1.007)	0.416		
DL_CO_	0.989 (0.978–0.999)	0.031	0.982 (0.971–0.993)	0.001
TLC	0.990 (0.977–1.002)	0.108		
UIP-like pattern	1.642 (1.124–2.399)	0.010	1.458 (0.960–2.212)	0.077
PF-ILD	3.075 (2.107–4.486)	< 0.001	3.053 (2.066–4.512)	< 0.001
Steroid ± IM	0.958 (0.484–1.897)	0.902		

*CI* confidence interval, *PF* progressive fibrosing, *ILD* interstitial lung disease, *BMI* body mass index, *iNSIP* idiopathic nonspecific interstitial pneumonia, *HP* hypersensitivity pneumonitis, *RA* rheumatoid arthritis, *SSc* systemic sclerosis, *SJS* SjÖgren syndrome, *FVC* forced vital capacity, *DL*_*CO*_ diffusing capacity of the lung for carbon monoxide, *TLC* total lung capacity, *UIP* usual interstitial pneumonia, *IM* immunosuppressant

### Comparison of PF-ILD criteria

**Fig. 4:**
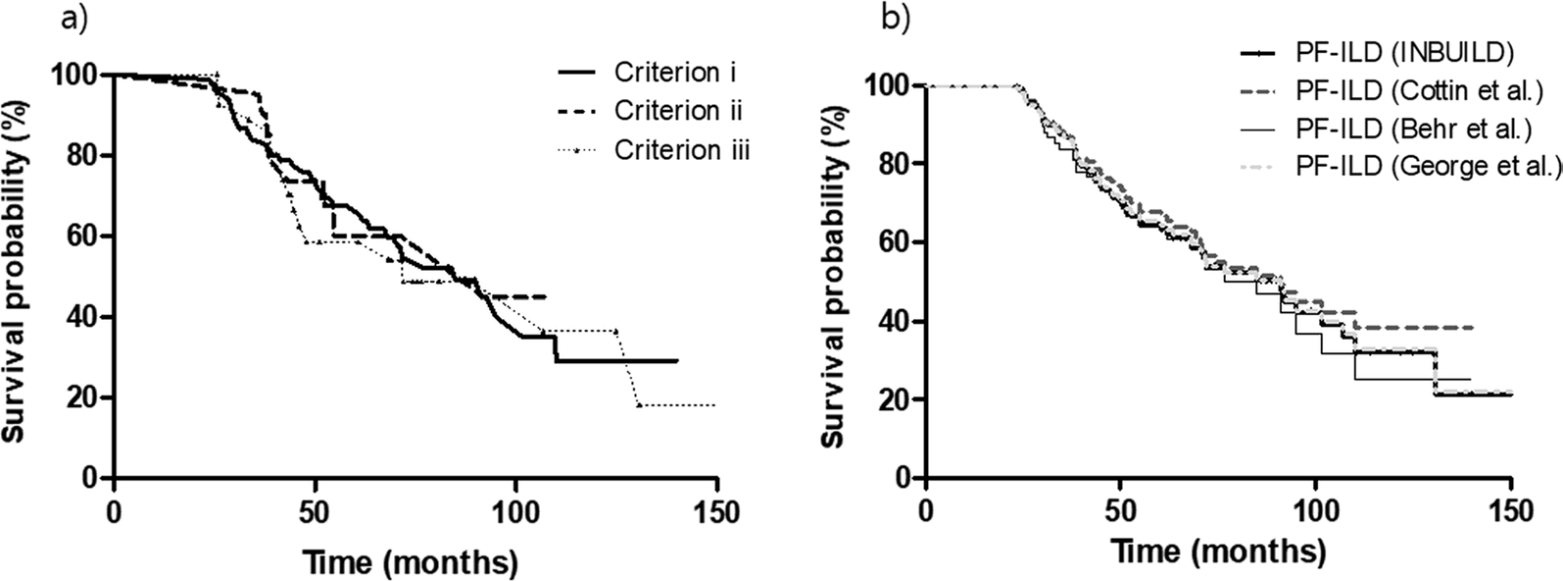
Comparison of survival curves in patients with PF-ILD according to PF-ILD diagnostic criteria. **a** Comparison of survival curves in patients with PF-ILD according to the diagnostic criteria used in this study. Criterion (i) a relative decline in FVC ≥ 10%; Criterion (ii) a relative decline in FVC of 5%–10% and worsening of respiratory symptoms or increased extent of fibrosis on HRCT; Criterion (iii) worsening of respiratory symptoms and increased extent of fibrosis on HRCT. **b** Comparison of survival curves in patients with PF-ILD according to the diagnostic criteria (INBUILD, Cottin et al., Behr et al., and George et al.)

We evaluated the prognostic influence of each PF-ILD criterion on survival. Survival was similar among PF-ILD patients defined by each criterion (*P* = 0.901, Fig. 4a).

The numbers of patients who met the PF-ILD criteria of the INBUILD trial and the studies of Cottin et al., Behr et al., and George et al. were 135 (34.1%), 125 (31.6%), 77 (19.4%), and 142 (35.9%), respectively. There was no significant survival difference between the four criteria (median survival time, 91.2 months [INBUILD]; 91.2 months [Cottin et al.]; 84.9 months [Behr et al.]; 91.2 months [George et al.]; *P* = 0.853) (Fig. 4b).

### Complications

During follow-up, 120 patients (30.3%) were hospitalised unexpectedly due to respiratory causes, and AE (61.7%) was the most common pulmonary-related cause of hospitalisation, accounting for 61.7% (median time from initial diagnosis to AE, 34.6 months). The PF-ILD group showed a significantly higher frequency of AE, pneumothorax, and PH than the non-PF-ILD group (Table 4).

**Table 4:** Comparison of complications between the PF-ILD and non-PF-ILD groups among patients with fibrosing ILD

	Total	PF-ILD	Non-PF-ILD	*P*-value
Number of patients	396	135	261	
Follow-up duration, months	68.9 ± 32.5	59.9 ± 28.4	73.5 ± 33.5	< 0.001
Unexpected respiratory hospitalisation	120 (30.3)	62 (45.9)	58 (22.2)	< 0.001
Acute exacerbation^a^	74 (18.7)	38 (28.1)	36 (13.8)	0.001
Time interval from diagnosis (months)	34.6 ± 28.2	29.3 ± 20.5	40.2 ± 33.9	0.037
Pneumonia	35 (8.8)	16 (11.9)	19 (7.3)	0.138
Pneumothorax	7 (1.8)	5 (3.7)	2 (0.8)	0.048
Pulmonary hypertension	89 (22.5)	45 (33.3)	44 (16.9)	< 0.001
Lung cancer	28 (7.1)	14 (10.4)	14 (5.4)	0.065

Data are expressed as mean ± standard deviation or number (%) unless otherwise indicated

^a^Acute worsening or development of dyspnea < 1 month in duration; new bilateral ground-glass opacity and/or consolidation superimposed on fibrosis; not fully explained by cardiac failure or volume overload

*PF* progressive fibrosing, *ILD* interstitial lung disease

## Discussion

In this study, approximately 34% of patients with fibrosing ILD other than IPF showed a progressive phenotype despite conventional management, and fibrotic HP and lower FVC were risk factors for PF-ILD. PF-ILD showed a poor outcome and was an independent prognostic factor for mortality in patients with non-IPF fibrosing ILD. In addition, although there are no uniformly accepted diagnostic criteria for PF-ILD, our results showed similar clinical outcomes among the previously proposed PF-ILD diagnostic criteria.

The proportion of patients who developed a progressive phenotype in our study was consistent with those in the previous literature, at approximately 30% [2, 10]. Moreover, decline in FVC of ≥ 10% was the most common criterion for PF-ILD in our study, consistent with previous reports [9, 10]. On the other hand, worsening symptoms and radiologic findings was the least common criterion in these studies. Nonetheless, a survival difference between the criteria was not observed in our study. A possible explanation for the lower proportion of this criterion is that changes in symptoms or radiologic findings are evaluated subjectively, as opposed to those of lung function, limiting determination of progression. In our study, the proportion of the criteria other than that of decline in FVC ≥ 10% were similar, accounting for 15–20% of the total.

In this study, fibrotic HP and lower FVC were risk factors for PF-ILD. When analysed according to ILD subtypes, lower lung function parameters in autoimmune ILD and HP were also associated with a progressive phenotype. Previous report also supports our results [24, 25]. In 266 patients with RA-ILD, Fu et al. reported that DL_CO_ less than 45% (OR, 8.31; 95% CI, 2.17–31.75; *P* < 0.001) and high titre of anti-cyclic citrullinated antibody (OR, 4.03; 95% CI, 1.04–15.69; *P* = 0.04) were risk factors for disease progression (a decrease in FVC > 10% predicted or DL_CO_ > 15% predicted) [24]. Similarly, in a study by Gimenez et al., lower FVC at baseline (HR, 1.03; 95% CI, 1.01–1.05, *P* = 0.003) was prognostic factor for mortality in 112 patients with chronic HP, along with decline in FVC ≥ 10% predicted after 6–12 months (HR, 4.13; 95% CI, 1.96–8.70, *P* = 0.005) [25]. These results show that advanced status in fibrosing ILD is associated with disease progression, and it was suggested that repetitive injury to the epithelium and vasculature forms a feed-forward loop circuit of increasing extracellular matrix and fibroblast activation, resulting in self-sustaining lung fibrosis [26].

In our study, the PF-ILD group showed poorer survival than the non-PF-ILD group. Recent reports also support our findings [7, 10]. Nasser et al., in an analysis of 165 patients with PF-ILD (autoimmune ILD, 46.7%; unclassifiable ILD, 31.5%; fibrotic HP, 8.5%; idiopathic interstitial pneumonia [IIP], 7.3%; other, 6.1%), reported that the 1 and 5 year survivals were 99.3 and 72%, respectively [10]. Brown et al., in 331 patients with PF-ILD (HP, 26.9%; autoimmune ILD, 26.6%; iNSIP, 18.4%; unclassifiable ILD, 15.1%; other ILD, 13.0%), also reported that 52-week mortality was 5.1%, similar to that of IPF (7.8%) [7]. However, the 5-year survival for PF-ILD in our study was lower (64.5% vs. 72.0%) than that of the previous study [10]. Considering that a larger proportion of patients with RA-ILD (42.4% vs. 4.2%) were included in our study, the survival difference might be due to the difference in composition of the ILD subtypes. However, regardless of the type of ILD, the prognosis of PF-ILD was poor in our study.

In this study, PF-ILD was an independent prognostic factor in patients with fibrosing ILD, along with age, and lung function at the time of diagnosis, but ILD subtype was not significant. Our findings are consistent with previous reports [27, 28]. Jegal et al., in an analysis of 179 patients with fibrotic IIP (IPF, 131; iNSIP, 48), revealed that after 6 months of follow-up, the only independent prognostic factors were changes in FVC, initial DLco, and sex, with no prognostic information conferred by the histologic diagnosis, suggesting the importance of disease progression [27]. Latsi et al., in an analysis of 104 patients with fibrotic IIP (IPF, 63; fibrotic NSIP, 41), also reported that serial trends in DL_CO_ were the only prognostic determinant after 1 year of follow-up, but histopathologic diagnosis was not [28]. These results suggest that disease progression after diagnosis is one of the most significant factors determining the outcome of fibrosing ILD.

The prevalence and survival of PF-ILD according to the various diagnostic criteria were similar in our study. The criteria of the INBUILD trial and Cottin et al. were similar except for the condition of changes in DL_CO_ used by Cottin et al. [9, 21]. George et al. also proposed integrated criteria for the diagnosis of PF-ILD [23]. However, the number of patients with PF-ILD as defined by Behr et al. [22] was smaller than that defined by other criteria because of the narrower definition using only lung function decline. Further validation of diagnostic criteria for PF-ILD should be performed in future studies.

There are some limitations to our study. First, this was a retrospective observational study performed at a single centre, and this may limit the generalisability of our results. The proportion of ILD subtype was also different from previous studies [9, 10, 22]. Moreover, difference in genetic susceptibility to PF-ILD between various population may lead to different results. However, the baseline patient characteristics in this study were similar to those in previous studies [9, 10]. Second, patients with unclassifiable ILD were not included in the study. However, unclassifiable ILD is diagnosed when specific diagnoses are impossible due to discordant clinical, radiological, and histopathologic information, or because patients are unable to undergo surgical lung biopsy due to high risk [29]. Therefore, the heterogeneous population and clinical courses of unclassifiable ILD may lead to inconsistent results [30]. Third, the definition of PF-ILD used in this study is not a consensus definition. Therefore, studies using different definitions may yield different results from ours. However, our study showed similar survival outcomes regardless of PF-ILD diagnostic criteria [10, 21–23].

## Conclusion

In conclusion, a progressive phenotype was identified in about 34% of patients with non-IPF fibrosing ILD. Our results suggest that in patients with fibrosing ILD, fibrotic HP and lower FVC confer a higher risk of PF-ILD, which has poor outcomes similar to those of IPF, regardless of the diagnostic criteria used.

## Supplementary Information

**Additional file 1: Figure S1.** Comparison of survival curves between the PF-ILD and non-PF-ILD groups among patients with fibrosing ILD according to each ILD subtype. **Table S1.** Risk factors for PF-ILD in patients with fibrosing ILD assessed using unadjusted logistic regression analysis according to ILD subtypes. **Table S2.** Prognostic factors for mortality in patients with fibrosing ILD assessed using an unadjusted Cox proportional hazard model according to ILD subtypes. **Table S3.** Prognostic factors for mortality in patients with PF-ILD assessed using a Cox proportional hazards model.

## Data Availability

The datasets used and/or analysed during the current study are available from the corresponding author on reasonable request.

## Abbreviations

CI: Confidence interval
HR: Hazard ratio
HRCT: High-resolution computed tomography
FVC: Forced vital capacity
HP: Hypersensitivity pneumonitis
IM: Immunosuppressant
iNSIP: Idiopathic nonspecific interstitial pneumonia
ILD: Interstitial lung disease
PF: Progressive fibrosing
PFT: Pulmonary function test
RA: Rheumatoid arthritis
SSc: Systemic sclerosis
SJS: SjÖgren syndrome
TLC: Total lung capacity
UIP: Usual interstitial pneumonia
