# Supplementary material for: Progressive fibrosing interstitial lung disease: prevalence and clinical outcome
Source: Respir Res. 2021 Oct 31;22:282. doi: 10.1186/s12931-021-01879-6 (PMC8559348; doi:10.1186/s12931-021-01879-6)
Supplement: Supplementary file 1 — Additional file 1: Figure S1. Comparison of survival curves between the PF-ILD and non-PF-ILD groups among patients with fibrosing ILD according to each ILD subtype. Table S1. Risk factors for PF-ILD in patients with fibrosing ILD assessed using unadjusted logistic regression analysis according to ILD subtypes. Table S2. Prognostic factors for mortality in patients with fibrosing ILD assessed using an unadjusted Cox proportional hazard model according to ILD subtypes. Table S3. Prognostic factors for mortality in patients with PF-ILD assessed using a Cox proportional hazards model. [file 12931_2021_1879_MOESM1_ESM.docx]

**Additional file 1**

Progressive fibrosing interstitial lung disease: prevalence and clinical outcome

Byoung Soo Kwon^1^, Jooae Choe^2^, Eun Jin Chae^2^, Hee Sang Hwang^3^, Yong-Gil Kim^4^, Jin Woo Song^5^

^1^Division of Pulmonary and Critical Care Medicine, Department of Internal Medicine, Seoul National University Bundang Hospital, Seongnam-Si, Gyeonggi-Do, Republic of Korea

^2^Department of Radiology, University of Ulsan College of Medicine, Asan Medical Centre, Seoul, Republic of Korea

^3^Department of Pathology, University of Ulsan College of Medicine, Asan Medical Centre, Seoul, Republic of Korea

^4^Department of Rheumatology, University of Ulsan College of Medicine, Asan Medical Centre, Seoul, Republic of Korea

^5^Department of Pulmonology and Critical Care Medicine, University of Ulsan College of Medicine, Asan Medical Centre, Seoul, Republic of Korea

**Additional file 1: Figure legend**


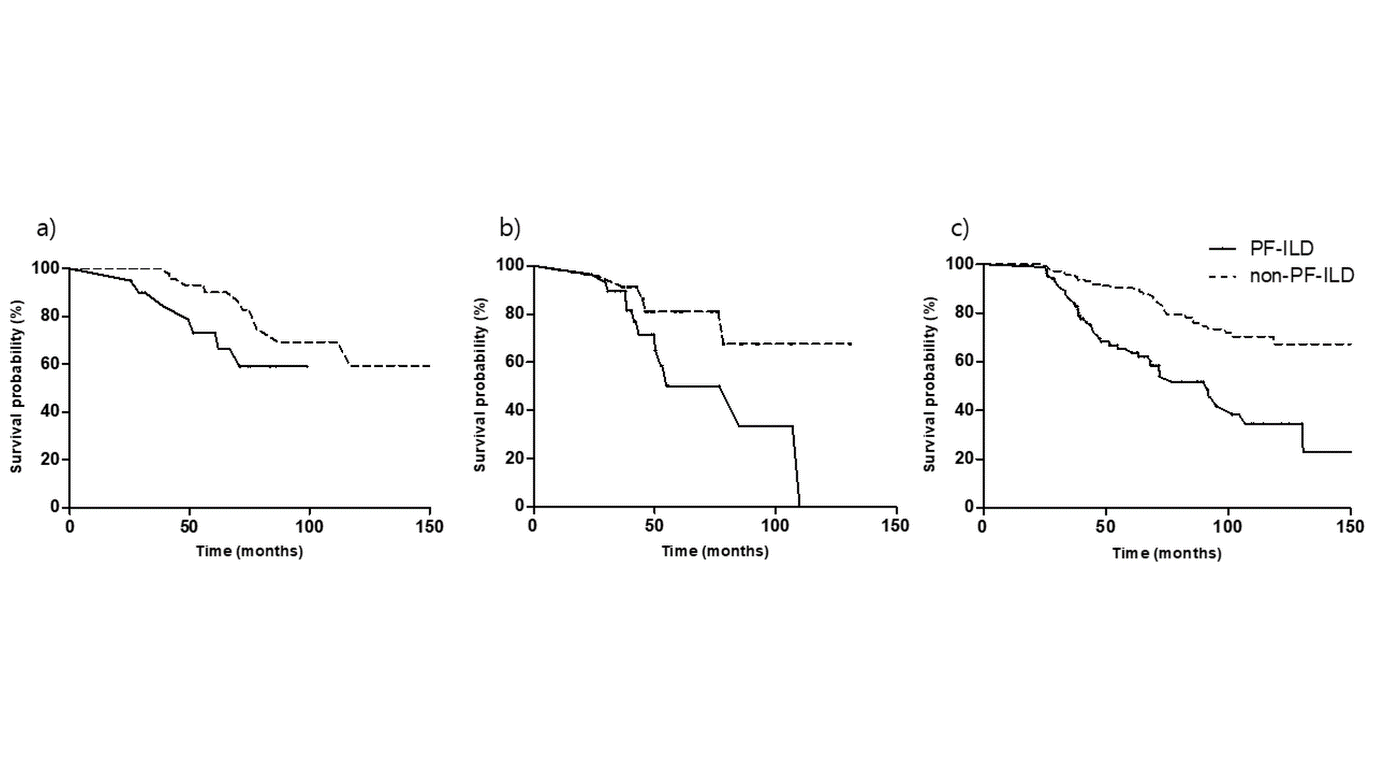


Additional file 1: Figure S1. Comparison of survival curves between the PF-ILD and non-PF-ILD groups among patients with fibrosing ILD according to each ILD subtype

a) Comparison of survival curves in patients with iNSIP

b) Comparison of survival curves in patients with fibrotic HP

c) Comparison of survival curves in patients with autoimmune ILDs

ILD, interstitial lung disease; iNSIP, idiopathic nonspecific interstitial pneumonia; HP, hypersensitivity pneumonitis

|  | Unadjusted | | | | | |
| --- | --- | --- | --- | --- | --- | --- |
|  | iNSIP | | HP | | Autoimmune ILD | |
|  | Odds Ratio (95% CI) | *P*-value | Odds Ratio (95% CI) | *P*-value | Odds Ratio (95% CI) | *P*-value |
| Age | 0.999 (0.949 – 1.050) | 0.955 | 1.012 (0.959 – 1.069) | 0.658 | 1.006 (0.982 – 1.031) | 0.623 |
| Male | 1.313 (0.471 – 3.659) | 0.603 | 0.495 (0.155 – 1.578) | 0.235 | 1.567 (0.929 – 2.644) | 0.092 |
| BMI | 1.126 (0.948 – 1.339) | 0.177 | 0.969 (0.844 – 1.111) | 0.649 | 1.059 (0.976 – 1.150) | 0.167 |
| Ever-smokers | 1.222 (0.439 – 3.406) | 0.701 | 0.414 (0.127 – 1.352) | 0.144 | 1.296 (0.771 – 2.182) | 0.328 |
| FVC | 1.002 (0.969 – 1.035) | 0.919 | 0.944 (0.900 – 0.989) | 0.015 | 0.984 (0.969 – 0.999) | 0.039 |
| DL_CO_ | 1.025 (0.989 – 1.062) | 0.180 | 0.974 (0.932 – 1.017) | 0.227 | 0.982 (0.968 – 0.996) | 0.013 |
| TLC | 1.008 (0.970 – 1.048) | 0.690 | 0.931 (0.879 – 0.986) | 0.014 | 0.975 (0.958 – 0.992) | 0.005 |
| UIP-like pattern | 2.111 (0.746 – 5.976) | 0.159 | 0.489 (0.141 – 1.694) | 0.259 | 0.959 (0.574 – 1.603) | 0.874 |
| Steroid ± IM | 1.075 (0.105 – 10.977) | 0.951 | 2.025 (0.309 – 13.275) | 0.462 | 3.225 (0.928 – 11.211) | 0.065 |

Additional file 1: Table S1. Risk factors for PF-ILD in patients with fibrosing ILD assessed using unadjusted logistic regression analysis according to ILD subtypes

PF, progressive fibrosing; ILD, interstitial lung disease; BMI, body mass index; iNSIP, idiopathic nonspecific interstitial pneumonia; HP, hypersensitivity pneumonitis; FVC, forced vital capacity; DL_CO_, diffusing capacity of the lung for carbon monoxide; TLC, total lung capacity; UIP, usual interstitial pneumonia; IM, immunosuppressant

Additional file 1: Table S2. Prognostic factors for mortality in patients with fibrosing ILD assessed using an unadjusted Cox proportional hazard model according to ILD subtypes

|  | Unadjusted | | | | | |
| --- | --- | --- | --- | --- | --- | --- |
|  | iNSIP | | HP | | Autoimmune ILD | |
|  | Hazard Ratio (95% CI) | *P*-value | Hazard Ratio (95% CI) | *P*-value | Hazard Ratio (95% CI) | *P*-value |
| Age | 1.052 (0.992 – 1.114) | 0.089 | 1.040 (0.985 – 1.099) | 0.155 | 1.079 (1.052 – 1.106) | <0.001 |
| Male | 2.712 (0.880 – 8.352) | 0.082 | 0.929 (0.322 – 2.679) | 0.891 | 1.297 (0.824 – 2.043) | 0.261 |
| BMI | 1.084 (0.927 – 1.268) | 0.311 | 0.945 (0.804 – 1.110) | 0.490 | 1.023 (0.953 – 1.098) | 0.527 |
| Ever-smokers | 1.426 (0.525 – 3.875) | 0.487 | 0.675 (0.230 – 1.987) | 0.476 | 1.253 (0.795 – 1.973) | 0.331 |
| FVC | 1.010 (0.980 – 1.041) | 0.516 | 0.972 (0.938 – 1.008) | 0.129 | 0.994 (0.981 – 1.007) | 0.372 |
| DL_CO_ | 1.003 (0.971 – 1.036) | 0.857 | 0.977 (0.940 – 1.015) | 0.236 | 0.987 (0.975 – 0.998) | 0.025 |
| TLC | 1.006 (0.970 – 1.043) | 0.743 | 0.929 (0.881 – 0.980) | 0.007 | 0.991 (0.976 – 1.005) | 0.216 |
| UIP-like pattern | 0.867 (0.320 – 2.352) | 0.780 | 1.976 (0.712 – 5.486) | 0.191 | 2.110 (1.300 – 3.424) | 0.002 |
| PF-ILD | 2.387 (0.876 – 6.505) | 0.089 | 2.774 (0.968 – 7.946) | 0.057 | 3.106 (1.976 – 4.883) | <0.001 |
| Steroid ± IM | 0.315 (0.071 – 1.407) | 0.130 | 25.352 (0.036 – 17816.513) | 0.334 | 0.875 (0.401 – 1.908) | 0.737 |

PF, progressive fibrosing; ILD, interstitial lung disease; BMI, body mass index; iNSIP, idiopathic nonspecific interstitial pneumonia; HP, hypersensitivity pneumonitis; FVC, forced vital capacity; DL_CO_, diffusing capacity of the lung for carbon monoxide; TLC, total lung capacity; UIP, usual interstitial pneumonia; IM, immunosuppressant

Additional file 1: Table S3. Prognostic factors for mortality in patients with PF-ILD assessed using a Cox proportional hazards model

|  | Unadjusted | | Multivariable | |
| --- | --- | --- | --- | --- |
|  | Hazard Ratio (95% CI) | *P*-value | Hazard Ratio (95% CI) | *P*-value |
| Age | 1.059 (1.026 – 1.093) | <0.001 | 1.072 (1.037 – 1.109) | <0.001 |
| Male | 1.056 (0.630 – 1.768) | 0.837 |  |  |
| BMI | 1.040 (0.967 – 1.119) | 0.287 |  |  |
| Ever-smokers | 0.929 (0.553 – 1.561) | 0.780 |  |  |
| ILD subtype |  | 0.100 |  |  |
| NSIP | 1 |  |  |  |
| HP | 1.581 (0.618 – 4.046) | 0.340 |  |  |
| RA-ILD | 1.590 (0.699 – 3.620) | 0.269 |  |  |
| SSc-ILD | 0.387 (0.080 – 1.865) | 0.237 |  |  |
| SJS-ILD | 0.805 (0.234 – 2.774) | 0.732 |  |  |
| FVC | 0.984 (0.968 – 1.001) | 0.070 | – | – |
| DL_CO_ | 0.987 (0.972 – 1.002) | 0.091 | 0.984 (0.967 – 1.000) | 0.049 |
| TLC^†^ | 0.978 (0.959 – 0.998) | 0.030 |  |  |
| UIP-like pattern | 1.536 (0.919 – 2.569) | 0.102 |  |  |
| Steroid ± IM | 3.703 (0.511 – 26.811) | 0.195 |  |  |

^†^TLC was excluded in the multivariable analysis due to close correlation with FVC (r=0.876, *P*<0.001)

CI, confidence interval; PF, progressive fibrosing; ILD, interstitial lung disease; BMI, body mass index; iNSIP, idiopathic nonspecific interstitial pneumonia; HP, hypersensitivity pneumonitis; RA, rheumatoid arthritis; SSc, systemic sclerosis; SJS, SjÖgren syndrome; FVC, forced vital capacity; DL_CO_, diffusing capacity of the lung for carbon monoxide; TLC, total lung capacity; UIP, usual interstitial pneumonia; IM, immunosuppressant
